# Postdischarge Follow-Up After Cardiac Hospitalizations Via Telehealth or In-Person: A Retrospective Cohort Study

**DOI:** 10.1016/j.jacadv.2025.101653

**Published:** 2025-03-14

**Authors:** Finlay A. McAlister, Luan Manh Chu

**Affiliations:** a The Division of General Internal Medicine, Faculty of Medicine & Dentistry, University of Alberta, Faculty of Medicine & Dentistry, University of Alberta, Edmonton, Alberta, Canada; b Alberta Strategy for Patient Oriented Research Support Unit, Edmonton, Alberta, Canada; c The Canadian VIGOUR Centre, University of Alberta, Edmonton, Alberta, Canada; d Provincial Research Data Services, Alberta Health Services, Edmonton, Alberta, Canada

**Keywords:** follow-up, outcomes, postdischarge

**What is the clinical question being addressed?**

Did the frequency of outpatient follow-up within 30 days of discharge after a cardiac hospitalization change during the COVID-19 pandemic, and were outcomes the same after telehealth visits compared to in-person follow-up visits during the pandemic?

**What is the main finding?**

During the pandemic, 30-day postdischarge access to care improved. Although in-person postdischarge visits were associated with fewer deaths or readmissions (adjusted HR [aHR]: 0.68 [95% CI: 0.61-0.76]), telehealth visits were not (aHR: 1.29 [95% CI: 1.14-1.46]), raising questions about whether telehealth confers the same benefits as in-person follow-up within the first 30 days after hospital discharge.

Early outpatient follow-up after hospitalizations for acute myocardial infarction or heart failure (AMI/HF) is associated with better outcomes and is a quality of care indicator.^1^ The frequency of outpatient visits for individuals with stable cardiovascular disease did not change after onset of the COVID-19 pandemic as the increase in telehealth visits offset in-person visit decreases.^2^ We designed this study to answer 2 questions: 1) did posthospitalization follow-up frequency change after onset of the pandemic; and 2) were outcomes the same after telehealth visits compared to in-person visits during the pandemic?

## Methods

Using administrative health care records, we examined all health care encounters (virtual and in-person) for adults in Alberta, Canada, between March 2019 and 2023; full details on methodology and case definitions have been previously published.^2^ Physician billing codes for telehealth visits were implemented in Alberta on March 17, 2020.

To answer the first question, we compared frequency of 30-day physician follow-up for patients who were discharged after AMI/HF hospitalization prepandemic (n = 10,310 between March 1, 2019, and February 29, 2020) vs those discharged between March 1, 2020, and February 28, 2023 (n = 22,763 without concomitant COVID-19). We only included the first hospitalization for each patient, calculated the Deyo-weighted Charlson comorbidity index based on all encounters in the prior 2 years, and conducted multivariable logistic regression to examine the association between time period (prepandemic vs pandemic) and 30-day follow-up (and outcomes) adjusting for patient sex, age, length of stay, acuity of admission, Charlson comorbidity score, and emergency department visits (LACE) score, Pampalon Material Deprivation Index, rural/urban residence, and discharge diagnosis.

To answer the second question, we only analyzed data for the 22,763 patients discharged after March 2020 without COVID-19 and used time-dependent covariates to capture the type and timing of physician follow-up after the hospital: no postdischarge visits in the first 14 days (referent group), ≥1 telehealth visit only, ≥1 in-person visit only, or 2 or more visits with mixed modalities (telehealth and in-person). Thus, we included all discharged patients and adjusted for potential differences in time to visits. Outcomes (30-day death/readmission) were analyzed using Cox proportional hazards models adjusted for age, sex, Pampalon deprivation index, rural vs urban residence, and the LACE score.^3^ We also compared 30-day outcomes after telehealth-only visits vs in-person-only visits using inverse probability of treatment weighting propensity score matching (using potential confounders as covariates: patient sex, age, LACE score, calendar year, Pampalon Material Deprivation Index, rural/urban residence, and discharge diagnosis). Balance was achieved as all standardized mean differences were <0.05 after matching. This study was approved by the University of Alberta Health Research Ethics Board.

## Results

Demographics, comorbidity profiles, and LACE scores of patients discharged after an AMI/HF hospitalization were similar to prepandemic and during the pandemic after exclusion of the 2,066 patients with concomitant COVID-19 (Table 1), although length of stay was about 1 day shorter during the pandemic. The frequency of 30-day follow-up was higher during the pandemic than prepandemic: 77.8% vs 73.4%, adjusted OR (aOR): 1.21 (95% CI 1.14-1.28), and mean number of visits 1.8 vs 1.5. During the pandemic, 16.7% of 30-day follow-ups were done by telehealth only, 48.3% in-person only, and 35.0% of patients had both types of visits within 30 days. In those without concomitant COVID-19, 30-day death or readmission after AMI/HF discharge was lower during the pandemic than prepandemic (13.6% vs 16.0%; aOR: 0.90 [95% CI: 0.84-0.96]).

**Table 1:** Characteristics and Outcomes for All Patients Discharged Alive After Hospitalization With Primary (Most Responsible) Diagnosis of Acute Myocardial Infarction or Heart Failure

	Prepandemic (March 1, 2019, to February 29, 2020) (N = 10,310)	Pandemic (March 1, 2020, to February 28, 2023) But Without COVID-19 (N = 22,763)	Pandemic (March 1, 2020, to February 28, 2023) But With Concomitant COVID-19 (N = 2,066)	Total (N = 35,139)
Age at admission, y	71.1 ± 14.3	70.0 ± 14.3	75.4 ± 13.4	70.6 ± 14.3
Female (%)	3,998 (38.8)	8,647 (38.0)	907 (43.9)	13,552 (38.6)
Rural (%)	2,019 (19.6)	4,347 (19.1)	365 (17.7)	6,731 (19.2)
Mean Charlson comorbidity score	3.2 ± 2.2	2.9 ± 2.0	4.0 (2.2)	3.1 (2.1)
Mellitus	4,328 ± 42.0	8,880 ± 39.0	1,067 ± 51.6	14,275 ± 40.6
HF	5,800 ± 56.3	11,377 ± 50.0	1,583 ± 76.6	18,760 ± 53.4
CAD	8,203 ± 79.6	17,808 ± 78.2	1,354 ± 65.5	27,365 ± 77.9
Hypertension	7,865 ± 76.3	17,144 ± 75.3	1,766 ± 85.5	26,775 ± 76.2
CKD (%)	3,676 ± 35.7	6,714 ± 29.5	1,059 ± 51.3	11,449 ± 32.6
Length of stay for index hospitalization, d	8.6 ± 13.3	7.6 ± 10.9	11.9 ± 18.7	8.2 ± 12.3
Mean LACE score	11.7 ± 3.1	11.2 ± 3.1	13.1 ± 2.9	11.5 ± 3.1
Outpatient follow-up within 30 d with a primary care physician or cardiologist/internal medicine specialist physician, any modality	7,568 (73.4)	17,702 (77.8)	1,467 (71.0)	26,737 (76.1)
Telehealth only	14 (0.1)	2,959 (13.0)	323 (15.6)	3,296 (9.4)
In-person only	7,512 (72.9)	8,564 (37.6)	673 (32.6)	16,749 (47.7)
Number of outpatient visits within 30 d with any physician and any modality	1.5 ± 1.4	1.8 ± 1.7	1.6 ± 1.7	1.7 ± 1.6
Outpatient follow-up within 30 d with cardiologist/internal medicine specialist physician, any modality	2,547 (24.7)	6,062 (26.6)	473 (22.9)	9,082 (25.8)
30-day death or readmission (overall)	1,644 (16.0)	3,089 (13.6)	519 (25.1)	5,252 (15.0)
30-day death or readmission (if had outpatient follow-up)	913 (12.1)	1,898 (10.7)	314 (21.4)	3,125 (11.7)
30-day death or readmission (if did not have outpatient follow-up)	731 (26.7)	1,191 (23.5)	205 (34.2)	2,127 (25.3)

Values are mean ± SD or n (%).

CAD = coronary artery disease; CKD = chronic kidney disease; HF = heart failure; LACE = length of stay, acuity of admission, Charlson comorbidity score, and emergency department visits.

Of the 22,763 patients discharged after March 2020 without COVID-19 (and with no visits being the referent group), those who had in-person postdischarge visits experienced fewer deaths or readmissions within 30 days (aHR: 0.68 [95% CI: 0.61-0.76] for in-person-only visits and aHR: 0.77 [95% CI: 0.70-0.86] for those with mixed modality visits), but those with telehealth-only visits had more deaths/readmissions (aHR: 1.29 [95% CI: 1.14-1.46]). The aOR for 30-day death/readmission after telehealth-only visits vs in-person-only visits was 1.65 (95% CI: 1.47-1.85) in the inverse probability of treatment weighting propensity score-matched analysis.

## Discussion

Our findings provide reassurance that the COVID-19 pandemic did not negatively impact 30-day follow-up or death/readmission after AMI/HF hospital discharge, at least in Alberta. This confirms the findings in 2 U.S. studies: one reported 30-day follow-up after all-cause hospital discharge increased from 72% prepandemic to 75% by December 2020^3^ and the other reported that 14% of postdischarge encounters were telehealth only by January 2021.^4^ Although neither of those studies examined 30-day outcomes, we found that 30-day death/readmission after AMI/HF hospital discharge was actually lower during the pandemic than prepandemic, after exclusion of those patients with concomitant COVID-19. However, as we cannot tell whether AMI/HF patients discharged during the pandemic were healthier than those discharged prepandemic (eg, if sicker pandemic-era patients hospitalized with AMI/HF were more likely to contract COVID-19 and/or die during the index hospitalization), we would encourage readers to adopt our conservative interpretation that the pandemic was at least not associated with worsening of 30-day outcomes after AMI/HF hospitalization in patients without concomitant COVID-19.

While we were able to confirm that in-person postdischarge visits during the pandemic were associated with an approximately one-quarter lower risk of death or readmission compared to no visits (consistent with prior literature),^1^ we found a negative association between telehealth-only follow-up visits and subsequent events. This is similar to an analysis of 16,987 emergency department (ED) discharges in Los Angeles in 2020 to 2021, which reported that the 30% of patients with telehealth-only visits post-ED were significantly more likely to return to the ED and be hospitalized than patients who received in-person follow-up visits within 30 days.^5^

The principal limitation of our study is that it is observational and thus can only be hypothesis-generating since there maybe multiple unmeasured confounders that influence both whether patients and/or physicians choose telehealth vs in-person visits and patient prognosis. Second, as we used administrative data, we were unable to adjust for severity of AMI/HF and also could neither assess the content of follow-up visits nor the quality of the care provided. Third, we do not know whether patients received unbilled follow-up visits through telephone calls or electronic messaging not captured by the physician claims dataset we used.

Although 30-day postdischarge access to care and outcomes for patients with AMI or HF were not negatively impacted by the pandemic, our findings raise questions about whether telehealth alone is adequate to assess clinical status in the first month after hospital discharge (when patients are at higher risk than stable outpatients).

## Funding support and author disclosures

The authors have reported that they have no relationships relevant to the contents of this paper to disclose.
